# DOA Estimation for Local Scattered CDMA Signals by Particle Swarm Optimization

**DOI:** 10.3390/s120303228

**Published:** 2012-03-07

**Authors:** Jhih-Chung Chang

**Affiliations:** Department of Information Technology, Ling Tung University, Taichung 408, Taiwan; E-Mail: changjc@mail.ltu.edu.tw; Tel.: +886-4-2389-2088; Fax: +886-4-3600-2535

**Keywords:** particle swarm optimization, direction-of-arrival estimation, CDMA, local scattering

## Abstract

This paper deals with the direction-of-arrival (DOA) estimation of local scattered code-division multiple access (CDMA) signals based on a particle swarm optimization (PSO) search. For conventional spectral searching estimators with local scattering, the searching complexity and estimating accuracy strictly depend on the number of search grids used during the search. In order to obtain high-resolution and accurate DOA estimation, a smaller grid size is needed. This is time consuming and it is unclear how to determine the required number of search grids. In this paper, a modified PSO is presented to reduce the required search grids for the conventional spectral searching estimator with the effects of local scattering. Finally, several computer simulations are provided for illustration and comparison.

## 1. Introduction

Adaptive array techniques have been developed for enhancing the performance of code-division multiple access (CDMA) systems. In the CDMA system, each user employs a unique pseudo-noise (PN) codeword to identify their data stream. Each user’s transmission interferes with all the other users and causes multiple access interference (MAI). The use of antenna arrays as a tool for improving coverage, reducing interference, and increasing capacity in CDMA systems has attracted significant interest [1]. Multiple propagation is common in cellular systems, which may result from local scatters in the vicinity of the sources. In macrocellular environments, high base station antennas will receive these locally scattered signals from the mobile terminal, which are coherent and confined to a narrow angular region [2]. In addition, the observation period is assumed to be short in comparison to the coherence time of the channel so that the channel may be modeled as time-invariant. A mobile terminal with local scattering can be well approximated by a single point source, as seen from the base station. The model can express the mobile and coherent interference from the surrounding environment under non line-of-sight conditions as another arithmetical model. In cellular radio systems, antenna array processing is considered to be a useful method to solve problems such as multipath and co-channel interference and increase capacity [1]. There have been some studies appraising the impact of a local scattering channel model on CDMA system, which propose uniform linear array (ULA) geometry [3]. They use a first-order Taylor expansion to linearize the nonlinear conventional array manifold produced by scatters and develop the generalized array manifold (GAM) model which can obtain better nominal DOA estimation for mobiles. Here, the multiple signal classification (MUSIC) searching function derived by the steering vector of the GAM model is termed as GMUSIC. However, in all these studies, estimation of direction of arrival (DOA) of the desired signals is required. Array outputs aligned with code-matched filter can make the DOA estimation of multiple sources equivalent to that for single source localization problem in a noisy environment. With the advantage of code-matched filter inherent in the CDMA system, it has been proved that the multiple signal classification estimator [4] can obtain an unbiased DOA estimation with low mean-square-error (MSE) [5]. It also contributes to solving the limitation that the number of array elements must be more than the number of impinging sources, but for a conventional spectral searching DOA estimator such as MUSIC, its searching complexity and estimating accuracy strictly depend on the number of search grids used during the search. This is time consuming and it is unclear how to determine the required number of search grids. Thus, in this paper, we will focus on the GAM model of ULA to employ the DOA estimation in multipath reflection scenario from scatters.

Particle swarm optimization (PSO) is a population-based stochastic optimization paradigm, in which each agent, named particle, of the population, named swarm, is thought of as a collision-proof bird and used to represent a potential solution [6]. Like a genetic algorithm (GA), PSO starts by initializing a population of random solutions and searches for optima by updating generations. But, PSO does not use any evolution operators. In PSO, particles fly through the problem space by following their own experience and the best experience attained by the swarm as a whole. In contrast to analytical or general heuristic methods, PSO is computationally efficient and has great capability of escaping local optima [7,8]. In addition, a key characteristic of PSO is that the algorithm itself is highly robust yet remarkably simple to implement, while processing similar capabilities as other evolutionary algorithms such as GA [9]. A maximum likelihood (ML) criterion with hard-constraint PSO based solution applied to DOA estimation is proposed in [10], that explores the potentially superior performance at less computational costs. The same PSO algorithm based on the ML methodology is also derived in [11], where the cost function is an extension of the ML criteria that were originally developed for angle estimation with some of the sensors that are perfectly calibrated, while others are uncalibrated. Recently an adaptive PSO algorithm has been successfully applied in ML optimization solutions [12]. In another report a combined fuzzy adaptive PSO and differential evolution are also used to solve economic dispatch problems [13]. In this paper, a modified adaptive inertia weight PSO (APSO) is proposed to rationally balance the global exploration and local exploitation abilities of the particle to the GMUSIC criteria function [3] for CDMA signals. The resulting estimator is called APSO-GMUSIC, where a simple and effective measure, individual search ability, is defined to indicate whether each particle lacks global exploration ability in each dimension. By employing APSO to treat our optimization problem of the desired signal direction angle *θ*. The DOA, obtained by the APSO-GMUSIC estimator, converges to the optimal or near optimal solution. Simulation results show that the proposed APSO-GMUSIC estimator is very suitable to treat the DOA estimation for CDMA signals in a local scattered scenario.

## 2. System Model

### 2.1. Array Data Model

Consider a DOA estimation scenario in a CDMA system with *P* users. The data bit *b_p_*(*t*) ∈ {−1, 1} of the *p*th user is spread by a pseudo-noise (PN) signature waveform *c_p_*(*t*) for *p* = 1, 2, …, *P*. The signature waveform of the *p*th user is composed of a spreading sequence of *L* chips, *i.e.*, $c_{p}(t)=\sum_{l=1}^{L}g_{p,l}\;p_{T_{c}}[t-(l-1)T_{c}]$, where $g_{p,l}\in \{-1/\sqrt{L},+1/\sqrt{L}\}$ is the *l*th spreading chip for the *p*th user and *p_Tc_*(*t*) is the spreading pulse of duration *T_c_*. Thus, the transmitted signal of the *p*th user during a bit interval *T_b_* can be represented by:

(1) $$s_{p}(t)=\varepsilon_{p}b_{p}(t)c_{p}(t)$$

where *ε_p_* is the signal amplitude. Let the processing gain of the spreading code be *L* = *T_b_*/*T_c_*. To simplify analysis, the PN code during a bit interval at a sample rate 1/*T_c_* can be expressed as an *L* × 1 vector of discrete sequence **c***_p_* = [*c_p_*_1_, *c_p_*_2_,…, *c_pL_*]*^T^* where the superscript *T* is the transpose operator.

Employing the code-matched filter and DOA estimation algorithm, we propose a receiving base station of CDMA communication system with a uniform linear array (ULA). The antenna array consists of *M* identical omidirectional elements and receives line-of-sight signals from *P* users that arrive at the array from different bearing angles *θ*_1_, *θ*_2_, …, *θ_p_* with respect to the broadside of array in a cell/sector. Assume that the array element space is *d*. The direction vector associated with the *p*th user is given by:

(2) $$a(\theta_{p})={\left[a_{1}(\theta_{p}),a_{2}(\theta_{p}),\ldots ,a_{M}(\theta_{p})\right]}^{T}$$

where *a_m_* (*θ_p_*) = exp[–*jπd*(*m*–1)sin(*θ_p_*)/*β*] denotes the response of the *m*th sensor array to a signal with unit amplitude arriving from the direction angle *θ_p_*, where *β* is the propagation speed of plane wave. Thus, the received baseband signal across the array can be written in vector form:

(3) $$x(t)=\sum_{p=1}^{P}s_{p}(t)a(\theta_{p})+n(t)$$

where $s_{p}(t)=\sum_{i=-\infty }^{\infty }\varepsilon_{p}b_{p}(i)c_{p}(t-{\mathrm{iT}}_{b}-\tau_{p})$, *τ_p_* is the time delay, and the observation noise vector **n**(*t*) represents the spatially and temporally zero mean white Gaussian noise vector.

### 2.2. MUSIC Algorithms for Local Scattering Channels

To show how the spatial signatures depend on the spatial distribution of the multipath propagation, we assume that the time dispersion produced by the multipath propagation is small compared to the reciprocal of the signal’s bandwidth, the time delay may be approximated as a phase shift $s_{p}(t-\tau_{\mathrm{ph}})≅e^{-j2\pi \;f_{c}\tau_{\mathrm{ph}}}s_{p}(t)$, where *f_c_* is the carrier frequency, and *τ_ph_* is the time delay associated with the *h*th scattered signal from the *p*th source arriving at the array. Let *s_p_*(*t*) be the signal scattered by the *p*th user. Due to the multipath propagation near the *p*th user, its contribution to the array is modeled as a superposition of *N_p_* scattered rays. Then, the data received at the array is given by *M* × 1 vector form:

(4) $$x(t)=\sum_{p=1}^{P}\sum_{h=1}^{N_{k}}\beta_{\mathrm{ph}}s_{p}(t)e^{-j\;2\pi \;f_{c}\tau_{\mathrm{ph}}}a_{\mathrm{ph}}(\theta_{p}+{\tilde{\Delta }}_{\mathrm{ph}})+n(t)$$

where the reflection coefficient *β_ph_* is the complex amplitude of the *h*th scattered signal from the *p*th user, **a***_ph_* is the response vector of the *h*th scattered signal from the *p*th user, and $j=\sqrt{-1}$. Δ̃*_ph_* is spread angle of the *p*th user due to local scattering. It is assumed that the power is normalized so that all rays have equal power 1/N*_p_*. Then $\beta_{\mathrm{ph}}=e^{j\;\chi_{\mathrm{ph}}}/\sqrt{N_{p}}$, where *χ_ph_* is i.i.d. and uniform over [0, 2*π*] [3]. Then, the received data vector **x**(*t*) can be expressed as:

(5) x(t)=Us(t)+n(t)

where **U** = [**u**_1_, **u**_2_, …, **u**_p_] and **s**(*t*) = [*s*_1_(*t*), *s*_2_(*t*), …, *s*_p_(*t*)]*^T^*. The *p*th column of **U**, which is denoted as **u***_p_*, represents the spatial signature associated with the signal *s_p_*(*t*), where $u_{p}=\sum_{h=1}^{N_{p}}\gamma_{\mathrm{ph}}\;a_{\mathrm{ph}}$ and $\gamma_{\mathrm{ph}}=\beta_{\mathrm{ph}}e^{-j\;2\pi \;f_{c}\tau_{\mathrm{ph}}}$. In order to pick out the *p*th user’s signal, a code-matched filter containing the specified PN code vector **c***_p_* is applied to **x**(*t*). For simplicity, the bit sampling index is dropped to make the equations more readable in the following description. Therefore, the *p*th user’s despread and sampled array vector signal **y***_p_* can be represented as:

(6) $$y_{p}={\mathrm{xc}}_{p}={\mathrm{Ls}}_{p}\;u(\theta_{p})+\sum_{q=1,\;q\neq p}^{P}s_{q}u(\theta_{q})+n_{p}$$

where *s_p_* = *ε_p_b_p_* and *s_q_* = *ε_p_b_p_κ_qp_*. Hence, *π_p_* = *E*{|*s_p_*|^2^} and *π_q_* = *E*{|*s_q_*|^2^} denote the average power of the desired *p*th user and each interferer user, respectively. The notation *E*{•} is used to denote the expectation operator. *κ_qp_* is the inner product of two different PN code vectors **c***_q_^T^***c***_p_*, and the *M* × 1 vector **n***_p_* with zero mean and variance *π*_n_ is the code-matched filter output of the noise vector **n**. It was shown in Appendix A of [3] that *κ_qp_* approaches a real Gaussian random variable with zero mean and unit variance for the normalized PN codes. Consequently, the signals of the undesired users through the code-matched filter, *i.e.*, MAI, can be viewed as a noise vector **n***_MAIp_* with zero mean and variance *π_MAIp_* and $n_{{\mathrm{MAI}}_{p}}=\sum_{q=1,\;q\neq p}^{P}s_{q}u(\theta_{q})$. The correlation matrix of Equation (6) in the observation interval is given by:

(7) $$R_{p}=E\{y_{p}y_{p}^{H}\}=L^{2}\pi_{p}u(\theta_{p})u^{H}(\theta_{p})+\pi_{\mathrm{np}}I_{M}$$

where the subscript *H* denotes conjugate transpose, *π_np_* = *π_MAIp_* + *π_n_*, and **I***_M_* is an *M* × *N* identity matrix.

For high angular resolution subspace methods, the MUSIC algorithm is a kind of DOA estimation technique based on eigenvalue decomposition (EVD), which is also called the noise subspace-based method. The eigendecomposition of Equation (7) can be expressed as:

(8) $$R_{p}=\sum_{m=1}^{M}\eta_{m}e_{\mathrm{pm}}e_{\mathrm{pm}}^{H}=\eta_{p1}e_{p1}e_{p1}^{H}+E_{\mathrm{pn}}\Lambda_{\mathrm{pn}}E_{\mathrm{pn}}^{H},$$

where *η*_*p*1_ ≥ *η_p_*_2_ = *η_p_*_3_ = ⋯ = *η_pM_* = *π_np_* are the eigenvalues of **R***_p_* and **e***_pm_* denotes the eigenvector associated with *η_pm_* for *m* = 1, 2, …, *M*. Moreover, **e***_p_*_1_ and **E***_pn_* = [**e***_p_*_2_, **e***_p_*_3_, ⋯, **e***_pM_*] are orthogonal and span the signal and noise subspace corresponding to **R***_p_*, respectively. **Λ***_pn_* = *π_np_* **I***_M_*_−1_ is the noise eigenvalue matrix. Furthermore, **e***_p_*_1_ spans the same signal subspace as that spanned by **a**(*θ_p_*). Thus, we have $E_{\mathrm{pn}}^{H}a(\theta_{p})=0$ and $a^{H}(\theta_{p})E_{\mathrm{pn}}=0$. The MUSIC estimates the DOA of the *p*th user from the highest peak of the following spectrum [3]:

(9) $$S_{p}(\theta )=\underset{\theta }{\text{max}}\frac{1}{\left|a^{H}(\theta )E_{\mathrm{pn}}E_{\mathrm{pn}}^{H}a(\theta )\right|},\;p=1,2,\cdots ,P$$

where the largest maxima of *S_p_*(*θ*) is taken to be the estimate of the DOA of the *p*th user.

As the angular spread Δ̃*_ph_* of the *h*th scattered ray from the *p*th source arriving at the array is relatively small, a first-order Taylor expansion of $u_{p}=\sum_{h=1}^{N_{p}}\gamma_{\mathrm{ph}}\;a_{\mathrm{ph}}$ in Equation (4) may be approximated as follows [14]:

(10) $${\tilde{u}}_{p}≅a_{p}+\rho_{p}\;d_{p}$$

where $\rho_{p}=\sum_{h=1}^{N_{p}}\gamma_{\mathrm{ph}}{\tilde{\Delta }}_{\mathrm{ph}}/\sum_{h=1}^{N_{p}}\gamma_{\mathrm{ph}}$ and **d***_p_* = ∂**a***_p_*/∂*θ_p_* is the first derivative of the steering vector. Equation (10) is a linear model and regards as the generalized array manifold (GAM) model with scattering, which is the superposition of the *p*th source steering vectors and their derivatives. Then, the received data vector **x̃**(*t*) can be expressed as:

(11) $$\tilde{x}(t)=\tilde{U}s(t)+n(t)$$

Therefore, the *p*th user’s despread and sampled array vector signal **ỹ***_p_* can be represented as:

(12) $${\tilde{y}}_{p}={\tilde{x}c}_{p}={\mathrm{Ls}}_{p}\tilde{u}(\theta_{p})+\sum_{q=1,\;q\neq p}^{P}s_{q}\tilde{u}(\theta_{q})+n_{p}$$

The correlation matrix of Equation (12) in the observation interval is given by:

(13) $${\tilde{R}}_{p}=E\{{\tilde{y}}_{p}{\tilde{y}}_{p}^{H}\}=L^{2}\pi_{p}\tilde{u}(\theta_{p}){\tilde{u}}^{H}(\theta_{p})+\pi_{\mathrm{np}}I_{M}$$

The eigendecomposition of Equation (13) can be expressed as:

(14) $${\tilde{R}}_{p}=\sum_{m=1}^{M}\eta_{m}{\tilde{e}}_{\mathrm{pm}}{\tilde{e}}_{\mathrm{pm}}^{H}=\eta_{p1}{\tilde{e}}_{p1}{\tilde{e}}_{p1}^{H}+{\tilde{E}}_{\mathrm{pn}}\Lambda_{\mathrm{pn}}{\tilde{E}}_{\mathrm{pn}}^{H},$$

With the GAM model, the MUSIC searching function of Equation (9) can be expanded into a MUSIC with local scattering. The MUSIC searching function derived by the steering vector of the GAM model in Equation (10), which is termed as GMUSIC and can be expressed as:

(15) $${\mathrm{GS}}_{p}(\theta ,\rho )=\underset{\theta ,\rho }{\text{max}}\frac{1}{\left|\xi^{H}{\bar{A}}^{H}(\theta ){\tilde{E}}_{\mathrm{pn}}{\tilde{E}}_{\mathrm{pn}}^{H}\bar{A}(\theta )\xi \right|},\;p=1,\;2,\;\cdots ,P$$

where **A̅**(*θ*) = [**a**(*θ*) **d**(*θ*)] and **ξ** = [1 *ρ*]*^T^*. The parameter of *ρ* can be estimated by using the method of [3]. And, the **Ẽ***_pm_* in Equation (15) is the noise subspace, which is the eigendecomposition of the autocorrelation matrix derived by the linearized steering vector in Equation (10). We can estimate DOA by searching the array manifold for direction vector and the largest peak of the function *GSp*(*θ*, *ρ*) denotes the DOA of the *p*th user.

### 2.3. Problem Formulation

Recently, a much more computationally efficient and more accurate parameter estimating approach via polynomial rooting method has been proposed to improve the spectral searching approaches for reducing the computational load. Due to the fact the scanning vector **A̅**(*θ*) of the GMUSIC is not Vandermonde structure, *GSp*(*θ*, *ρ*) cannot be implemented by using the polynomial rooting approach. The computational complexity of the classical subspace approach of MUSIC estimator is about 12*M*^3^ complex multiplications (CM) for computing EVD of a *M* × *M* dimension matrix. Let the search number be *B*. Therefore, the total required CM for computing Equation (15) of the GMUSIC estimator is about 12*M*^3^ + *BM*^2^. The performance of the abovementioned spectral searching approach is governed by the scanning grid size and the number of search grids while implementing the high-resolution DOA estimation. A major problem of GMUSIC type algorithms is the heavy computational load incurred with spatial spectral search when a smaller grid size is adapted. However, it is time consuming and the optimum search grid size is not clear. Smaller grid size can improve estimate accuracy, but the required computational load also becomes relatively larger (e.g., if grid sizes are set to 1°, 0.1° and 0.01°, then the required search numbers *B* = 181, 1,801, and 18,001, respectively.). With the number of particle population *P_N_* and the maximum number of iterations *k*_max_, the computational complexity of our APSO approach is about *P_N_* × *k*_max_. Hence, the computational complexity of APSO is smaller than the GMUSIC. Therefore, in order to reduce computational load, this paper investigates the feasibility of applying the PSO to replace the spectrum searching approach. As a result, the proposed PSO-based searching GMUSIC estimator does not increase the computational complexity, but significantly reduces the searching process requirement compared with the spectrum searching GMUSIC.

## 3. The Proposed PSO Based Searching Algorithms

Due to the fact that the performance of the abovementioned spectral searching GMUSIC estimator is governed by the scanning grid size and the number of search grids while implementing the high-resolution DOA estimation, it is time consuming and the search grid is not clear. In this section, we present PSO based searching approaches by maximizing the fitness function of GMUSIC at each iteration.

### 3.1. HPSO-GMUSIC Estimator

In order to reduce the scanning accurate angle problems of high computation cost, we use the PSO to replace scan in the ULA. For time-space signal processing, each particle can be treated as a point in the *P*-dimensional problem space and a swarm consisting of *D* random particles and then searches for best position (solution) by updating generations until exceeding the limit of iteration number. The position of the *i*th particle is represented as **θ***_i_* = [θ*_i_*_1_, θ*_i_*_2_, …, θ*_iP_*], the rate of the position change (velocity) for *i*th particle is represented as **v***_i_* = [v*_i_*_1_, v*_i_*_2_, …, v*_iP_*]. The best previous position of the *i*th particle, which gives the best fitness value, is recorded as **p***_i_* = [p*_i_*_1_, p*_i_*_2_, …, p*_iP_*]. And, the index of the best particle among all the particles in the population is represented by **p***_g_* = [p*_g_*_1_, p*_g_*_2_, …, p*_gp_*] and called global best location. In every iteration, the local bests and global bests are determined through evaluating the fitness values of the current population of particles. Two factors characterize a particle status on the search space: its position and velocity. The *P*-dimensional position for the *i*th particle in the *k*th iteration can be denoted as **θ***_i_*(*k*) = [**θ***_i_*_1_(*k*), **θ***_i_*_2_(*k*), …, **θ***_i_*_P_(*k*)]. Similarly, the velocity for the *i*th particle in the *k*th generation can be described as **v***_i_*(*k*)= [v*_i_*_1_(*k*), v*_i_*_2_(*k*) …, v*_iP_*(*k*)]. The velocity and the position of the *i*th particle at the (*k* + 1)th iteration for *i* = 1, 2, …, *D* and *k* = 1, 2, …, *k*_max_ are updated according to the following equations:

(16) $$v_{i}(k+1)=w(k)v_{i}(k)+c_{1}r_{1}(k)⊗(p_{i}(k)-\theta_{i}(k))+c_{2}r_{2}(k)⊗(p_{g}(k)-\theta_{i}(k))$$

(17) $$\theta_{i}(k+1)=\theta_{i}(k)+v_{i}(k+1)$$

where ⊗ denotes element-wise product and the positive acceleration constants *c*_1_ and *c*_2_ are two positive constants named learning factors. **r**_1_(*k*) and **r**_1_(*k*) are *P*-dimensional vectors consisting of independent random numbers uniformly distributed between 0 and 1, which are used to stochastically vary the relative pull of **p***_i_* and **p***_g_* in order to simulate the unpredictable component of natural swarm behavior. The inertia weight *w*(*k*) based on the linear decreasing strategy is considered critically for the convergence behavior of PSO [10], which is selected to decrease during the optimization process. Thus, PSO tends to have more global search ability at the beginning of the run while having more local search ability near the end of the optimization. Given a maximum value *w*_max_ and a minimum value *w*_min_, *w*(*k*) is updated as:

(18) $$w(k+1)=w_{\text{max}}-\frac{w_{\text{max}}-w_{\text{min}}}{k_{\text{max}}}k$$

where *k*_max_ is the maximum number of iterations, *w*_max_ and *w*_min_ are chosen as 0.9 and 0.4 respectively in this work as in [10]. Because the PSO particle represents a series of priorities that range from −90° to 90°, all parameters of the *P*-dimensional particle positions, either initialized or updated during search, must be confined within [*θ*_min_, *θ*_max_] = [−90°, 90°], avoiding infeasible particle positions that can lead to slow PSO searches. The new particle position is calculated using Equation (17).

A particle position vector is converted to a candidate solution vector in the problem space through a suitable mapping. The score of the mapped vector evaluated by a GMUSIC function is regarded as the fitness of the corresponding particle. For time-space signal processing, the DOA GMUSIC estimation problem in this paper is to find the *i*th particle position or estimate angle *θ_ip_*(*k*) of the *p*th user to maximize the following fitness function:

(19) $${\text{fitness}}_{\mathrm{ip}}(k)=\frac{1}{\xi^{H}{\bar{A}}^{H}(\theta_{\mathrm{ip}}(k)){\tilde{E}}_{\mathrm{pn}}{\tilde{E}}_{\mathrm{pn}}^{H}\bar{A}(\theta_{\mathrm{ip}}(k))\xi },\;p=1,\;2,\;\cdots ,P$$

At the end of iteration, global best location *p_gp_* (*k*_max_) is the final estimated angle defined as *θ̂_p_* = *p_gp_*(*k*_max_). The final global best position **p***_g_* (*k*_max_) is taken as the GMUSIC estimates of users. As a result, the PSO with linear decreasing inertia weight, particle velocity limitation, and particle position clipping is termed as hard-constraint PSO GMUSIC (HPSO-GMUSIC). Unfortunately, the HPSO-GMUSIC is not so facile to implement for its overhead on computing the search space mapping, particle velocity limitation and particle position clipping. In this paper, we present a more feasible and efficient modified PSO algorithm for the HPSO-GMUSIC estimator.

### 3.2. The Proposed Multiple Adaptive Inertia Weight

The inertia weight is critical for the performance of PSO, which balances global exploration and local exploitation abilities of the swarm. In other words, the inertia weight *w*(*k*) is employed to control the impact of the previous history of velocities on the current velocity, thereby influencing the trade-off between global and local exploration abilities of the “flying points”. A large inertia weight tends to facilitate searching new area and global exploration. Conversely, a small inertia weight facilitates local exploitation in the current search area. Suitable selection of the inertia weight provides a balance between global and local exploration abilities and thus requires less iteration on average to find the optimum. During the search every particle dynamically changes its position, so every particle locates in a complex environment and faces different situation. Therefore, each particle along every dimension may have different trade off between global and local search abilities. According to [15], it has been shown that the performance of the PSO algorithm with linearly decreasing inertia weight has the ability to quickly converge, but the PSO may lack global search ability at the end of a run and may fail to find the required optima in cases when the problem to be solved is too complicated and complex. But to some extent, this can be overcome by employing a self-adapting strategy for adjusting the inertia weight. In this subsection, inertia weight is dynamically adapted for every particle along every dimension. A measure, individual search ability, which characterizes the faced situation for every particle is defined. Basing on this measure, the particle could decide to whether to increase or decrease the values of inertia weight by means of the transfer function. The fine strategy of dynamically adjusting inertia weight could lead to improvement in performance of PSO.

For velocity updating, the last two parts of Equation (16) can be viewed as the accelerations parts and can be defined as *a_ip_*(*k*) = *c*_1_·*rand*(•)·(*p_ip_*(*k*) – *θ_ip_*(*k*)) and *a_gp_*(*k*) = *c*_2_·*rand*(•)·(*p_ip_*(*k*) – *θ_ip_*(*k*)), where *rand*(•) is independent random number with uniformly distributed between [0,1]. So we could consider that the particle is moving with velocity of *v_ip_*(*k*) and acceleration of *a_ip_*(*k*) and *a_gp_*(*k*). But, *a_gp_*(*k*) is the dominant term for improving convergence rate. Suppose that the mass of the *i*th particle in the *p*th dimension is normalized to 1 kg. According to the principle of mechanics, *a_gp_*(*k*) = *f_ip_*(*k*), where *f_ip_*(*k*) is an outside force, which is put on the particle comes from the “pulling” of *p_ip_*(*k*) and *p_gp_*(*k*). For ULA, the DOA is a one-dimensional searching problem. In order to make the particle fly towards optimal region quickly, *v_ip_*(*k*) should turn to the direction of *f_ip_*(*k*) as soon as possible. Define the error between *p_ip_*(*k*) and *θ_ip_*(*k*) is *z_ip_*(*k*) = *p_gp_*(*k*) – *θ_ip_*(*k*). Let *m_ip_*(*k*) = |*z_ip_*(*k*)|/*θ*_max_ which is a number between 0 and 1. The velocity update problem of the *i*th particle on the *p*th dimension can be divided into two classes:

1. Firstly, *v_ip_*(*k*) and *z_ip_*(*k*) are in the same direction, *z_ip_*(*k*)/*v_ip_*(*k*) ≥ 0. If *m_ip_*(*k*) is relatively large, it means the particle is in the right direction, but the velocity is too small. Therefore, the particle needs to speed up, and the inertia weight *w_ip_*(*k*) needs to be set larger. If *m_ip_*(*k*) is relatively small, it means the particle has come to the location, that is near the optimal region. So the velocity of this particle should slow down and the neighborhood of the state should be searched carefully, and *w_ip_*(*k*) should be set smaller.
2. On the other hand, consider *v_ip_*(*k*) and *z_ip_*(*k*) on different direction, *z_ip_*(*k*)/*v_ip_*(*k*) < 0. If *m_ip_*(*k*) is relatively large, it means that the particle’s state is far from the optimal region. So the particle needs to change its velocity as soon as possible, and the inertia weight *w_ip_*(*k*) needs to be set smaller. If *m_ip_*(*k*) is relatively small, it means that it’s not urgent for the particle to change its direction on this dimension, and *w_ip_*(*k*) could be set a large value.

Based on the aforementioned analysis, an adaptive inertia weight strategy is proposed and is shown in Table 1. The individual normalized search ability of the *i*th particle along the *p*th dimension is defined as *m_ip_*(*k*). It is noted that the value of *w_ip_*(*k*) is a function of *m_ip_*(*k*). To get a balance of global search and local search ability, *w_ip_*(*k*) cannot be too large or too small, thus *w_ip_*(*k*) is limited in the range of [*w*_min_, *w*_max_], which is like the process of normalization. Therefore, we used *μ-law* algorithm to achieve our strategy for every particle along every dimension, normalization of the *m_ip_*(*k*) to *w̅_ip_*(*k*). The *μ-law* algorithm is a companding scheme used in telephone network [16]. Increasing the value of *u*, the dynamic range capability of *μ-law* can be improved and defined by:

(20) $${\bar{w}}_{\mathrm{ip}}(k)=\frac{\text{log}(1+\mu m_{\mathrm{ip}}(k))}{\text{log}(1+\mu )},\;0\leq m_{\mathrm{ip}}(k)\leq 1$$

where *m_ip_*(*k*) and *w̅_ip_*(*k*) are normalized input and output values respectively, and the positive constant *μ* is the compression parameter. The reciprocal slop of this curve is given by the derivative of *m_ip_*(*k*) with respect to *w̅_ip_*(*k*). For case 1, the value for small *m_ip_*(*k*) increases at the expense of the value for large *m_ip_*(*k*). To accommodate these conflicting requirements (*i.e.*, a reasonable values for both small and large *m_ip_*(*k*)), a compromise is usually made in choosing the value of parameter *μ* for *μ-law*. The requirement of case 2 is opposite. Finally, based on our strategy and Equation (20), we can define multiple adaptive inertia weight *w_ip_*(*k*) as:

(21) $$w_{\mathrm{ip}}(k)=\{\begin{array}{ll} w_{\text{min}}+{\bar{w}}_{\mathrm{ip}}(k), & \frac{z_{\mathrm{ip}}(k)}{v_{\mathrm{ip}}(k)}\geq 0\\ w_{\text{min}}+(1-{\bar{w}}_{\mathrm{ip}}(k)), & \frac{z_{\mathrm{ip}}(k)}{v_{\mathrm{ip}}(k)}<0 \end{array}$$

**Figure 1.**
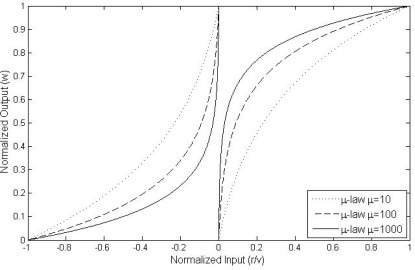
Normalized output *w̅_ip_*(*k*) *versus* normalized input *z_ip_*(*k*) / *θ*_max_ under *w*_min_ = 0.

**Figure 2.**
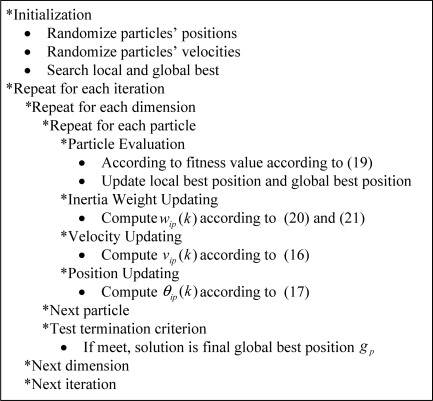
Flowchart illustrating the main steps of the APSO-GMUSIC.

In Equation (21), *w*_min_ be added to avoid particles from stopping moving. The curves of *w_ip_*(*k*) with *w*_min_ = 0 using different *μ* can be plotted in Figure 1. Then, we also investigate the sensitivity for APSO-GMUSIC with different values of *μ* However, it accords with our strategy for different *μ*. Thus, *μ*-law algorithm with *μ* = 100 is chosen. Note that for every particle in population, *w_ip_*(*k*) is unique and can be computed individually. Therefore, the single inertia weight *w_ip_*(*k*) can be replaced by a multiple adaptive inertia weight *w_ip_*(*k*). The proposed APSO-GMUSIC seems to be robust to control parameters due to the intrinsic advantages of the algorithm and the separation of the problem-independent PSO kernel from newly introduced problem-specific features in our design for adaptive multiple inertia weight. Finally, the steps for implementing the APSO-GMUSIC are shown in Figure 2 and described in the list that follows.

## 4. Computer Simulations

Several computer simulations for illustration and comparison are presented in this section. We use a 6-element ULA with half wavelength for simulation. Consider an asynchronous CDMA system with PN codes of length 31 and BPSK modulation. The spatial signature **u***_p_* is generated by *N_p_* = 30 independent identically distributed local scatters with uniform random angular spread of width 2Δ̃*_p_*. It is assumed that Δ̃*_p_* is uniformly distributed over the interval [−Δ*p*, Δ*_p_*]. Each scatter has equal power and is randomly, uniformly distributed over [0, 2*π*]. The value of parameter *ρ* is assumed to be known. Eight users are impinging on the array with random impinging angles of uniform distribution in the interval [−90°, 90°]. All signal powers are set to be 20 dBW. The additive background noise is assumed to be white Gaussian distribution with zero-mean and unit Watts of power. For GMUSIC, the search resolution (grid) is set to 1°, 0.1°, and 0.01° under the search range of [−90°, 90°]. The root-mean-squared error (RMSE) is calculated in an average manner as $\text{RMSE}={\left[(1/\mathrm{FP})\sum_{j=1}^{F}\sum_{p=1}^{P}{\left({\hat{\theta }}_{\mathrm{jp}}-\theta_{p}\right)}^{2}\right]}^{0.5}$, where *F* indicates the number of independent simulation runs, *P* is the number of users, *θ̂_jp_* is the *p*th estimate DOA achieved in the *j*th run, and *θ_p_* is the true DOA of the *p*th user. The PSO parameters chosen for all the experiments are summarized in Table 2. All PSO algorithms start with random initializations and are terminated if the maximum iteration *k*_max_ is reached or the global best particle position is not updated in 20 successive iterations. Every simulation result is presented after 200 data bits were processed and it is averaged by *F* = 10^3^ independent Monte Carlo runs with independent noise samples for each run.

**Figure 3.**
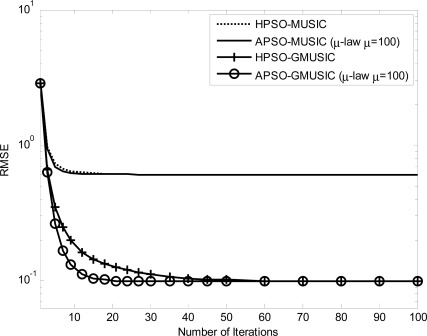
DOA RMSE *versus* the number of iterations.

**Figure 4.**
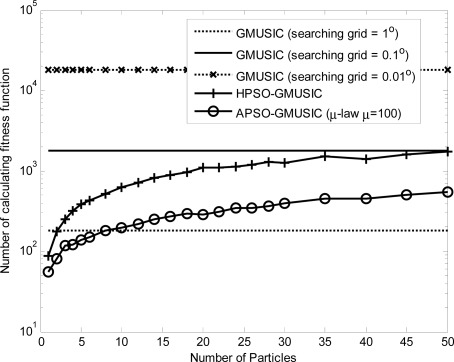
The required number of calculating fitness function (*B*) *versus* number of particles.

**Figure 5.**
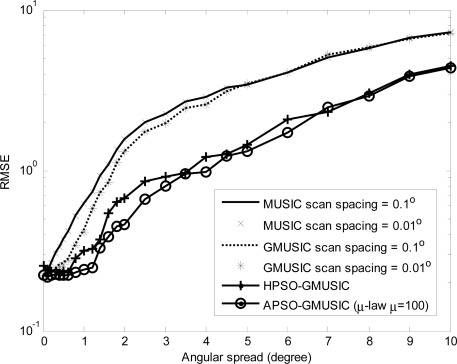
RMSE of DOA estimation *versus* angular spread.

**Figure 6.**
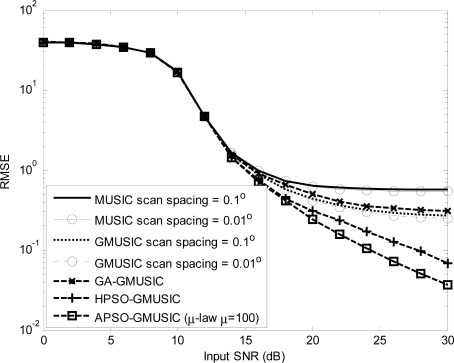
RMSE of DOA estimation *versus* the input SNR of desired user for 2Δ*_p_* = 1°.

**Figure 7.**
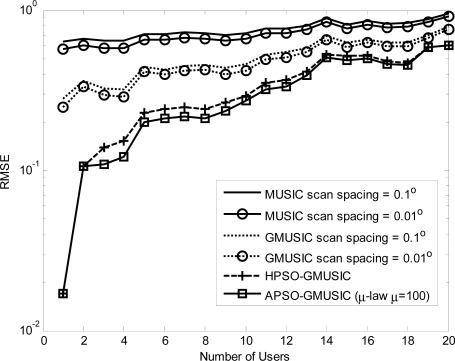
RMSE of DOA estimation *versus* the number of users for SNR = 20 dBW and 2Δ*_p_* = 1°.

Comparison results with other estimators, including the GMUSIC, HPSO-GMUSIC and APSO-GMUSIC with *μ* = 100 to DOA estimation error are presented. Figure 3 depicts the convergence in terms of DOA RMSE *versus* the number of iterations. As a result, the HPSO-GMUSIC requires more iterations to achieve convergence. Note that the proposed APSO-GMUSIC achieves fast convergence with the selected parameters, which means that it needs less iterations to approach the desired global extreme. Figure 4 shows the required number of calculating fitness function (*B*) *versus* number of particles. For the number of particles in the population, more particles may increase success in searching for optima due to sampling state space more thoroughly. However, more particles require more evaluation cost. The HPSO-GMUSIC needs more particles to approach the desired global extreme. It is confirmed that the proposed PSO-based searching approaches can reduce the computational complexity of the GMUSIC due to the searching process. As expected, this figure also provides a great improvement over the convergence rate on optimization problems. In fact, additional adaptive multiple inertia weight operation can improve the searching speed and RMSE performance further. Figure 5 presents the RMSE of DOA estimation *versus* varying angular spreads. We note that the subspace-based techniques show serious degradation when faced with local scatters. Local scattering may be viewed as a form of model error and gives rise to the perturbation of the noise subspace. Again, these figures show that the proposed APSO-GMUSIC method yields significantly superior performance over the other methods in the presence of local scatters. For comparison, the result of GA-GMUSIC estimator is also provided. The same parameters of GA-GMUSIC estimator are used in [17]. Figure 6 shows the RMSE *versus* different SNR of the desired user for angular spreads 2Δ*_p_* = 1°. For the low SNR case, all of methods may produce highly biased estimates. Clearly, with the compatible searching resolution, the APSO-GMUSIC can save the required number of searching grids and improve the RMSE performance, as compared with the other estimators. The GA-GMUSIC has a poor performance than HPSO-GMUSIC and APSO-GMUSIC. It is well known that premature convergence degrades the performance of GA and reduces the search ability [18]. In addition, it has been shown that the performance of the PSO algorithm with linearly decreasing inertia weight has the ability to quickly converge, the PSO may lack global search ability at the end of a run and may fail to find the required optima in cases when the problem to be solved is too complicated and complex [19]. But to some extent, this can be overcome by employing the proposed adaptive multiple strategy for adjusting the inertia weight. Finally, in Figure 7, we compare the RMSE performance against the number of active user *P*, given SNR = 20 dBW and angular spread 2Δ*_p_* = 1°. Basically, the RMSE is increased quite steadily with the increase of *P*. It can be observed that the APSO-GMUSIC obtain more performance improvement when the number of users *P* is reasonably increasing. Among them, the proposed APSO method achieves the lowest RMSE.

## 5. Conclusions

In this paper, we have presented a PSO based searching DOA estimation method, named APSO, which uses the GMUSIC searching function for CDMA signals. With the code-matched filter, the MAIs after code-decorrelation appear as noises for CDMA signals. The rooting MUSIC method is suboptimal in the presence of the noise and MAI and the GMUSIC cannot be implemented by using the polynomial rooting approach. However, the proposed techniques reduce the required search grids for the conventional spectral searching estimators. In the previous work on DOA estimation using PSO algorithm [10], hard constraints have been taken, during each iteration of PSO algorithm. In this paper, multiple inertia weight has been incorporated in PSO. In conjunction with a modified PSO for angle searching, the proposed approach can reduce the required computational load for the conventional spectral searching MUSIC estimator with local scattering. Moreover, the convergence of the proposed approach is much faster. Computer simulations have demonstrated the effectiveness of the proposed approach. Furthermore, a common drawback with MUSIC-like technique is that it is a suboptimal estimator and tends to suffer from low performance due to low SNR, small sample size, and correlated sources. Thus, how to develop a cheaper way and work well with correlated or even coherent sources will be important in the future work.

## Figures and Tables

**Table 1.** Adaptive inertia weight strategy.

Value of *w_ip_* (*k*)		Value of *m_ip_* (*k*)		
		Small	Middle	Large
Directions of *v_ip_* (*k*) and *z_ip_* (*k*)	Same	small	middle	large
	Opposite	large	middle	small

**Table 2.** Selected PSO Parameters.

	*c*_1_	*c*_2_	The number of particles	The number of iterations	Constraints	*w*_max_	*w*_min_	Inertia weight feature
HPSO	2	2	20	20	Hard constraint	0.9	0.4	Single and linearly
APSO						1.1	0.1	Multiple and used *μ–law*
